# Lipoxin A_4_ ameliorates lipopolysaccharide-induced lung injury through stimulating epithelial proliferation, reducing epithelial cell apoptosis and inhibits epithelial–mesenchymal transition

**DOI:** 10.1186/s12931-019-1158-z

**Published:** 2019-08-22

**Authors:** Jing-xiang Yang, Ming Li, Xin-ou Chen, Qing-quan Lian, Qian Wang, Fang Gao, Sheng-wei Jin, Sheng-xing Zheng

**Affiliations:** 1 Department of Anesthesia and Critical Care, The Second Affiliated Hospital and Yuying Children’s Hospital of Wenzhou Medical University, Zhejiang, 325027 China; 2 Birmingham Acute Care Research Group, Institute of Inflammation and Aging, University of Birmingham, Birmingham, B15 2TT UK

**Keywords:** Acute respiratory distress syndrome, Alveolar type II cells, Proliferation, Apoptosis, Epithelial to mesenchymal transition

## Abstract

**Background:**

Acute respiratory distress syndrome (ARDS) is characterized by alveolar epithelial disruption. Lipoxins (LXs), as so-called “braking signals” of inflammation, are the first mediators identified to have dual anti-inflammatory and inflammatory pro-resolving properties.

**Methods:**

In vivo, lipoxinA_4_ was administrated intraperitoneally with 1 μg/per mouse after intra-tracheal LPS administration (10 mg/kg). Apoptosis, proliferation and epithelial–mesenchymal transition of AT II cells were measured by immunofluorescence. In vitro, primary human alveolar type II cells were used to model the effects of lipoxin A_4_ upon proliferation, apoptosis and epithelial–mesenchymal transition.

**Results:**

In vivo, lipoxin A_4_ markedly promoted alveolar epithelial type II cells (AT II cells) proliferation, inhibited AT II cells apoptosis, reduced cleaved caspase-3 expression and epithelial–mesenchymal transition, with the outcome of attenuated LPS-induced lung injury. In vitro, lipoxin A_4_ increased primary human alveolar epithelial type II cells (AT II cells) proliferation and reduced LPS induced AT II cells apoptosis. LipoxinA_4_ also inhibited epithelial mesenchymal transition in response to TGF-β_1_, which was lipoxin receptor dependent. In addition, Smad3 inhibitor (Sis3) and PI3K inhibitor (LY294002) treatment abolished the inhibitory effects of lipoxinA_4_ on the epithelial mesenchymal transition of primary human AT II cells. Lipoxin A_4_ significantly downregulated the expressions of p-AKT and p-Smad stimulated by TGF-β_1_ in primary human AT II cells.

**Conclusion:**

LipoxinA_4_ attenuates lung injury via stimulating epithelial cell proliferation, reducing epithelial cell apoptosis and inhibits epithelial–mesenchymal transition.

**Electronic supplementary material:**

The online version of this article (10.1186/s12931-019-1158-z) contains supplementary material, which is available to authorized users.

## Background

Acute respiratory distress syndrome (ARDS), an acute inflammatory pulmonary process, causes intense and diffuse alveolar architecture damage and the development of interstitial and alveolar protein-rich edema, leading to acute hypoxemic respiratory failure [1, 2]. In ARDS, the alveolar epithelium is the primary target where cell damage occurs. The degree of alveolar epithelial damage can predict the outcome of ARDS [3, 4]. Consequently, the repair of the alveolar epithelium plays a crucial role in the resolution of ARDS [4]. Recent literatures have demonstrated that apoptosis of alveolar epithelial cells contributed to the loss of alveolar epithelial cells and the development of ARDS [5–7]. Inhibiting apoptosis has been shown to attenuate lung injury in animal models [6].

Epithelial-mesenchymal transition (EMT) is the process in which epithelial cells differentiate into mesenchymal (fibroblast-like) cells expressing mesenchymal biomarkers such as α-Smooth muscle actin (α-SMA), and N-cadherin [8]. The EMT was associated with lung injury and could lead to the prognosis of ARDS [9]. Furthermore, inflammation stimulated by HCL can also lead to EMT in HCL-induced ARDS models [10, 11]. Another study demonstrated that trichostatin A attenuated ventilation augmented-EMT playing a role in the reparative phase of ARDS [12]. Both EMT and apoptosis of the alveolar epithelium are crucial for the progression of ARDS.

Lipoxins (LXs), as so-called “braking signals” of inflammation, are endogenous lipid mediators derived from arachidonic acid [13]. They were the first mediators identified to have dual anti-inflammatory and inflammatory pro-resolving properties [14]. Lipoxin A4 (LXA4) was shown to inhibit neutrophil and eosinophil recruitment [15], promote macrophage clearance of apoptotic neutrophils [16], and increase survival in a rat CLP model [17]. Our previous studies showed that LXA4 inhibited inflammation following inhaled LPS-induced lung injury [18]. LXA4 increased alveolar fluid clearance in rat lung injury model [19], and LXA4 promoted alveolar epithelial repair by stimulating epithelial cell wound repair, proliferation, and reducing apoptosis in vitro [20].

The alveolar epithelial cells may either undergo apoptosis or EMT in ARDS. In this study, we aimed to investigate whether LXA4 could promote type II alveolar lung epithelial cells proliferation, whilst inhibiting apoptosis in vivo and in vitro. Furthermore, we also investigated if LXA4 inhibited EMT in vivo and reduced TGF-β_1_ induced EMT in human primary type II alveolar epithelial cells.

## Materials and methods

### Materials

LXA4 and LY294002 (PI3K inhibitor) were obtained from Cayman Chemical Company (Ann Arbor, MI, USA). LPS (*Escherichia coli* serotype 055: B5), Sis3 (smad3 inhibitor) and SP-C antibody were purchased from Sigma (St Louis, MO, USA), BOC-2 (N-t- BOC-PHE-LEU-PHE-LEU-PHE; Gene Script USA Inc., Piscataway, NJ, USA) and BML-111 (Enzo Life Sciences, NY, United States) were purchased from Shang Hai Bo Yun. Antibody against anti-alpha smooth muscle actin (α-SMA) antibody, Vimentin and the secondary antibodies were obtained from Abcam Company (Cambridge, UK). Antibodies against E-cadherin and N-cadherin were from Cell Signaling Technology Company (Boston, USA). Recombinant Human TGF-β_1_ (HEK293 derived) was purchased from Peprotech Company (Rocky Hill, USA). DMEM and FBS were purchased from Life Technologies BRL (Grand Island, NY). Protein levels were determined using a Bicinchoninic acid kit (Thermo Scientific).

### Primary human lung alveolar type II (HAT II) cell culture

Human alveolar type II (HAT II) cells were isolated from lungs of grossly normal appearance after lung tumor resection. The cells were isolated in accordance with approval from the local research ethics committees at the University of Wenzhou Medical University (Wen Zhou, China). Primary human AT II cells were extracted according to the methods described previously (see online supplement) [20].

### Stimuli and inhibitors

HAT II cells were treated with LXA4 (0, 0.1, 1, 10, 100 nM, Cayman Chemical Company, USA) with or without LPS (1 μg/ml, *Escherichia coli* serotype 055:B5). Appropriate vehicle controls were used for all experiments with inhibitors. Inhibitors were used at the following concentrations according to manufacturers’ instructions: LY294002, a PI3-kinase inhibitor (Calbiochem, Nottingham, UK); Sis3 (smad3 inhibitor), Boc-2 (N-t-Boc-Phe-Leu-Phe-Leu-Phe; GenScript USA Inc., the ALXR antagonist) and BML-111(Enzo Life Sciences, NY, United States, the ALXR agonist), all at 10 μM. Inhibitors were added to cells 30 min before every treatment.

### Animal model of ALI/ARDS

C57BL/6 J mice at 6–8 weeks of age were purchased from the Shanghai SLAC Laboratory Animal Co. Ltd. The animals were acclimatized for 7 days prior to experimental use. Mice were caged with free access to food and fresh water in a temperature-controlled room (22–24 °C) on a 12-h light/dark cycle. Mice (male; ethics code: 2015048) were randomized into 5 groups of 6 mice per group: control group, LPS group (24 h, 48 h, 72 h), LPS + LXA4 group. For the induction of ARDS, mice were anaesthetised and instilled by intra-tracheal (IT) route as a model of direct lung injury with LPS (10 mg/kg dissolved in 30ul N.S) for 24 h, 48 h or 72 h. No treatment control mice were anaesthetised and instilled by intra-tracheal (IT) route with physiological saline. In LPS + LXA4 group, LXA4 was administrated by intraperitoneal injection at 1 μg/per mouse 10 min after intra-tracheal (IT) LPS administration. Mice were subsequently sacrificed by using cervical dislocation, lungs were removed and washed with sterile PBS and stored in 4% paraformaldehyde for HE and immunofluorescence, or at − 80 °C for Western blot, in tube for wet/dry ratio.

### Immunofluorescence

Lung tissue were fixed and stained as the method described in the online Supplementary Information.

### Quantitative real-time PCR and reverse transcriptase-PCR

Total RNA samples in HAT II cells were isolated using TRIzol reagent (Invitrogen, Carlsbad, California, USA) according to the manufacturer’s protocol. The cDNA of mRNA was synthesized using the reverse transcription kit (Bio-Rad, USA). The expression of mRNA was detected using SYBR green super-mix PCR kit (Bio-Rad) by qPCR (ABI7500, Applied Biosystems). The gene-specific primers used are listed in Table 1 and mRNA normalized to GAPDH, was calculated using the 2^-ΔΔCt^ method.

**Table 1:** Real-time PCR templates and primers used for gene manipulation

Gene symbol	Forward primer (5′-3′)	Reverse primer (5′-3′)
SNAIL	CCTCTCACTGGGTCTTCTGG	GGTCTTCTTCCGCTCCTCTC
AQP5	GCTGCCATCCTTTACTTCTACC	GGTCTTCTTCCGCTCTTCC
FIBRONECTIN	CCAAGCAGGAGTCAAACGAG	TCTTCCATCTCACGCATCTG
α-SMA	CCGACCGAATGCAGAAGGA	ACAGAGTATTTGCGCTCCGAA
CDH1	GGTCTCTCTCACCACCTCCA	CCTCGGACACTTCCACTCTC
CDH2	CGTGAAGGTTTGCCAGTGT	CAGCACAAGGATAAGCAGGA
SP-C	CCTTCTTATCGTGGTGGTGGT	TCTCCGTGTGTTTCTGGCTCAT
GAPDH	GACAACAGCCTCAAGATCATCAG	ATGGCATGGACTGTGGTCATGAG

### Protein extraction and Western blot analysis

Cells or lung sections were washed in ice-cold PBS and harvested using RIPA buffer supplemented with protease inhibitors. The resulting supernatant fraction was homogenized in 1x SDS–PAGE sample buffer and boiled for 5 min at 99 °C. For the immunoblotting, protein lysates were electrophoresed via 10% SDS-PAGE gel and then transferred to to polyvinylidene difloride membranes. Membranes were blocked and incubated with the indicated primary antibody (Ab) overnight at 4 °C. Bound primary Abs were incubated with appropriate secondary Abs for 1 h. The proteins were detected using chemiluminescence reagents (Thermo Scientific). Images were scanned with a UVP imaging system and analyzed using an Image Quant LAS 4000 mini system (GE Healthcare Bio-Sciences AB, Uppsala, Sweden).

### Flow cytometry (FCM)

Apoptosis of HAT II cells was assessed using flow cytometry. HAT II cells were left in serum free media for 24 h before exposure to LPS (1 μg/ml) with or without LXA4100nM for 24 h. After treatment with LPS and LXA4, HAT II cells were harvested and suspended in the binding buffer supplied in the Annexin V-FITC/Propidium iodide (PI) Apoptosis Detection Kit, and were then stained with Annexin V-FITC and Propidium iodide (PI) according to the manufacturer’s instruction (BD Biosciences, USA). The cytometric data were analyzed with FlowJo software.

### Blinding method

The present study adopted randomized, blinded methods. The randomization list of animals was computer-generated by the statistician using SAS/STAT software.

### Statistical analysis

Data are presented as mean ± SD or mean ± SEM. All data were analyzed using one-way ANOVA, followed by a Tukey test for post hoc comparisons. P < 0.05 was considered significant. Statistical analyses were performed using Prism 6.0 software (Graph Pad Software, San Diego, CA).

## Results

### LXA4 stimulates AT II cell proliferation and reduces AT II cell apoptosis in LPS induced lung injury

**Fig. 1:**
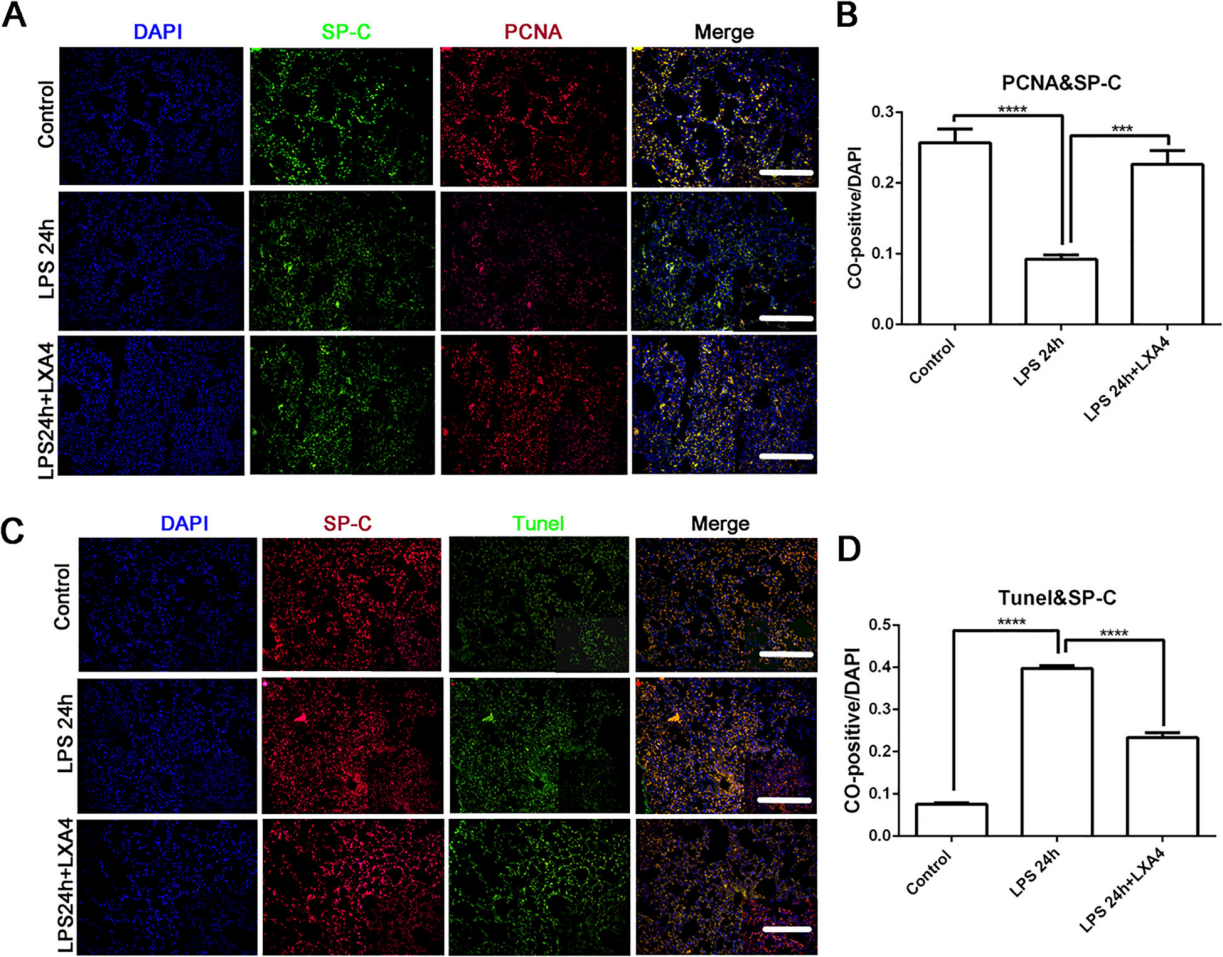
LXA4 stimulates AT II cell proliferation and reduces AT II cell apoptosis in LPS induced lung injury. C57BL/6 J mice were intra-tracheal given N.S or LPS 10 mg/kg for 24 h, with or without LXA4 1μg intraperitoneal injection per mouse. Immunofluorescence staining of lung specimens was shot by fluorescence microscope and calculated by positive goals compared to DAPI. **a**-**b**: co-dying of SP-C and PCNA (× 200, × 400). **c**-**d**: co-dying of SP-C and TUNEL (× 200, × 400), scar bar = 50 μm. Data were presented with means ± SD; n = 4; ***P < 0.001, ****P < 0.0001

As shown in Additional file 1: Figure S1, intratracheal instillation of LPS (10 mg/kg) in mice induced lung injury with characteristic neutrophil accumulation, septal thickening, interstitial fluid accumulation, and alveolar hemorrhage at 24 h (Additional file 1: Figure S1B), 48 h (Additional file 1: Figure S1C) and 72 h (Additional file 1: Figure S1D). Treatment with LXA4 attenuated LPS-induced lung injury (Additional file 1: Figure S1E). The lung injury score was consistent with the histopathological changes (Additional file 1: Figure S1F). The Wet/Dry (W/D) ratio increased after LPS treatment, and LXA4 reversed the W/D ratio induced by LPS at 72 h (Additional file 1: Figure S1G), suggesting that LXA4 can alleviate lung permeability damage induced by LPS. The proliferation and apoptosis of AT II cells in the intratracheal LPS murine model of ALI/ARDS were observed by lung specimen immunofluorescence double staining of SP-C (a type II cell marker) and PCNA, SP-C and TUNEL respectively. LPS inhibited the proliferation of AT II cell (SP-C/PCNA double positive cells) and LXA4 abrogated the inhibition of LPS on AT II cells proliferation at 24 h (Fig. 1a, b). Meanwhile, apoptosis of AT II cells was calculated by the co-dying of SP-C and TUNEL. As shown in Fig. 1c and d, LPS increased AT II cell apoptosis, and LXA4 reduced apoptosis of AT II cell induced by LPS at 24 h.

### LXA4 decreases LPS-stimulated caspase-3 activation in lung tissue

**Fig. 2:**
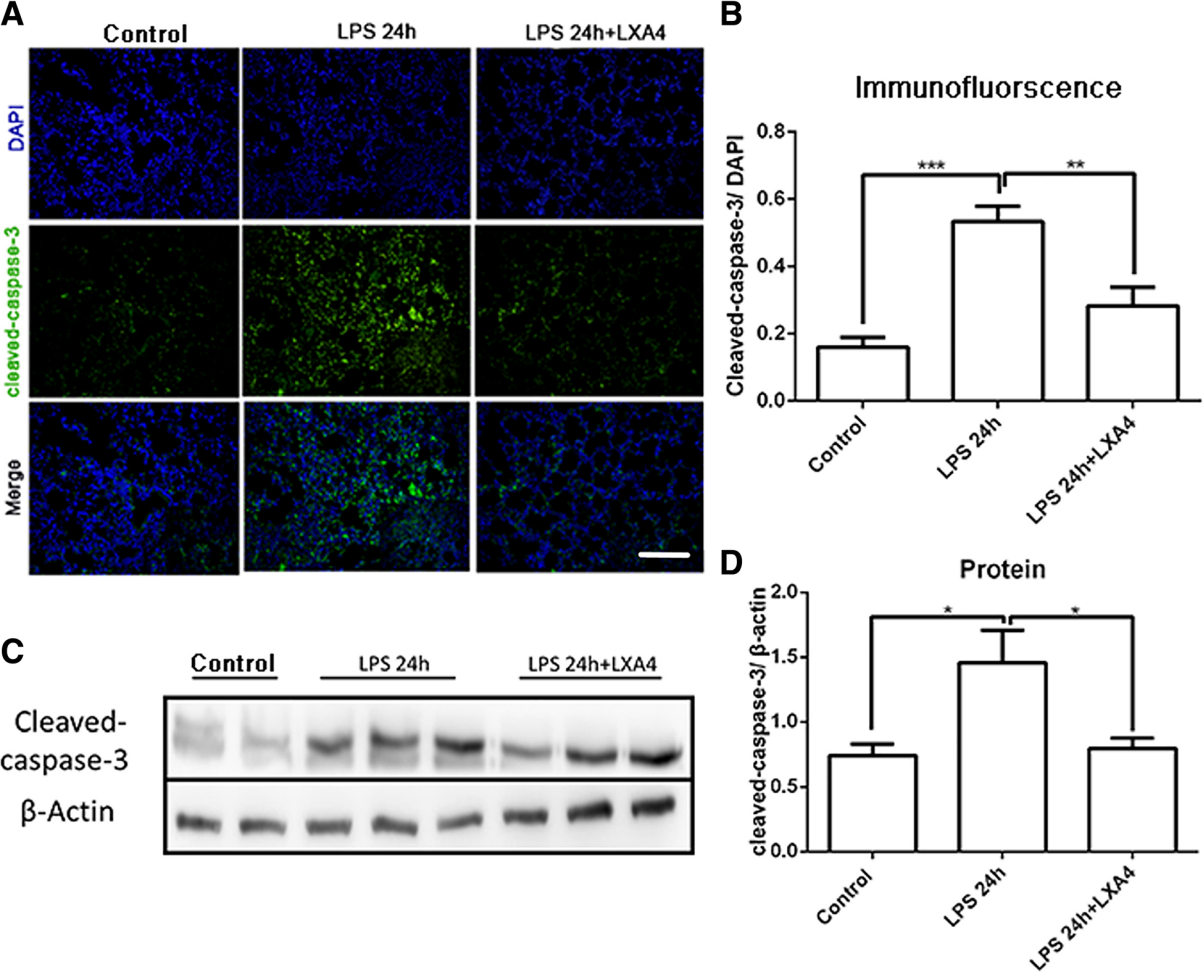
LXA4 alleviates apoptosis in LPS-induced lung injury. C57BL/6 J mice were intra-tracheal given N.S or LPS 10 mg/kg for 24 h, with or without LXA4 1μg per mouse intraperitoneal injection. Immunofluorescence staining of lung specimens was shot by fluorescence microscope and calculated by positive goals compared to DAPI. **a**-**b**: Immunofluorescence staining of cleaved-caspase-3, scar bar = 50 μm. **c**-**d**: WB of cleaved-caspase-3. Data were presented with means ± SD, *P < 0.05, **P < 0.01, ***P < 0.001; n = 3

Apoptosis is accompanied by caspase-3 cleavage, so cleaved caspase-3 was measured by both immunofluorescence and western blotting in different groups. Our results demonstrated that LPS increased the expression of cleaved caspase-3 in lung tissue, and LXA4 inhibited LPS-stimulated cleaved caspase-3 expression at 24 h in lung tissue (Fig. 2).

### LXA4 reduces HAT II cells apoptosis and stimulates primary human lung alveolar type II (HAT II) cells proliferation in vitro

**Fig. 3:**
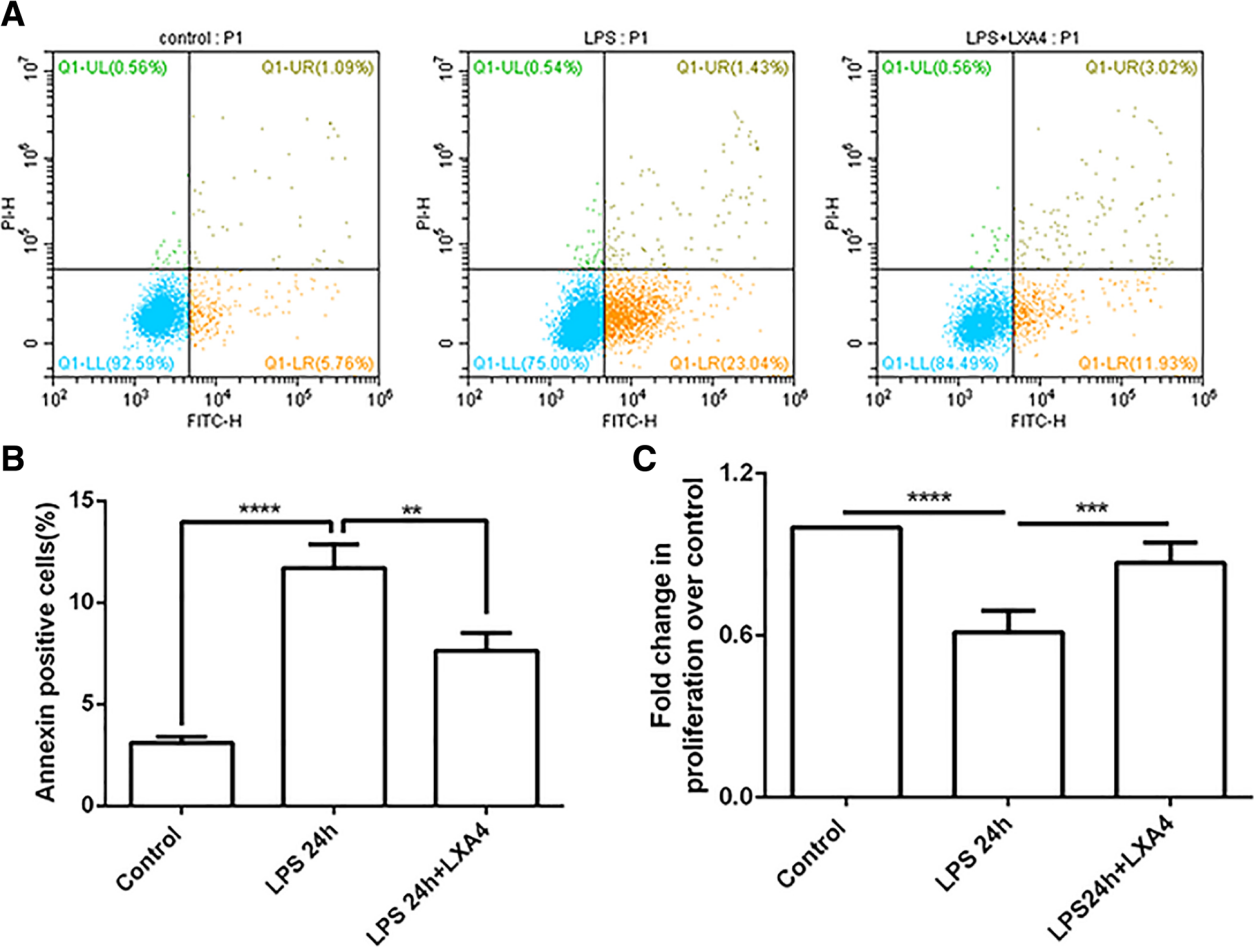
LXA4 stimulates HAT II cell proliferation and reduces HAT II cell apoptosis in vitro. HAT II cells were cultured as mentioned in the methods. **a** and **b**: apoptosis of HAT II after the stimulation of LPS and LXA4. **c**: Proliferation of HAT II after the stimulation of LPS and LXA4. Data were presented with means ±SEM. **P < 0.01, ***P < 0.001, **** *P* < 0.0001, n = 4 for each culture condition, repeated using cells from 4 donors

As shown in Fig. 1, LXA4 stimulated HAT II cell proliferation and reduced HAT II cell apoptosis in the intratracheal LPS murine model of ALI/ARDS. Next, we investigated whether LXA4 could also stimulate HAT II cell proliferation and reduce HAT II cell apoptosis in vitro. As shown in Fig. 3a and c, LPS increased HAT II cell apoptosis, and treatment with LXA4 reduced apoptosis of HAT II cell induced by LPS at 24 h. LPS inhibited the proliferation of HAT II cells, while LXA4 promoted their proliferation. (Fig. 3c).

### LXA4 inhibits the epithelial-mesenchymal transition (EMT) in LPS-induced lung injury

**Fig. 4:**
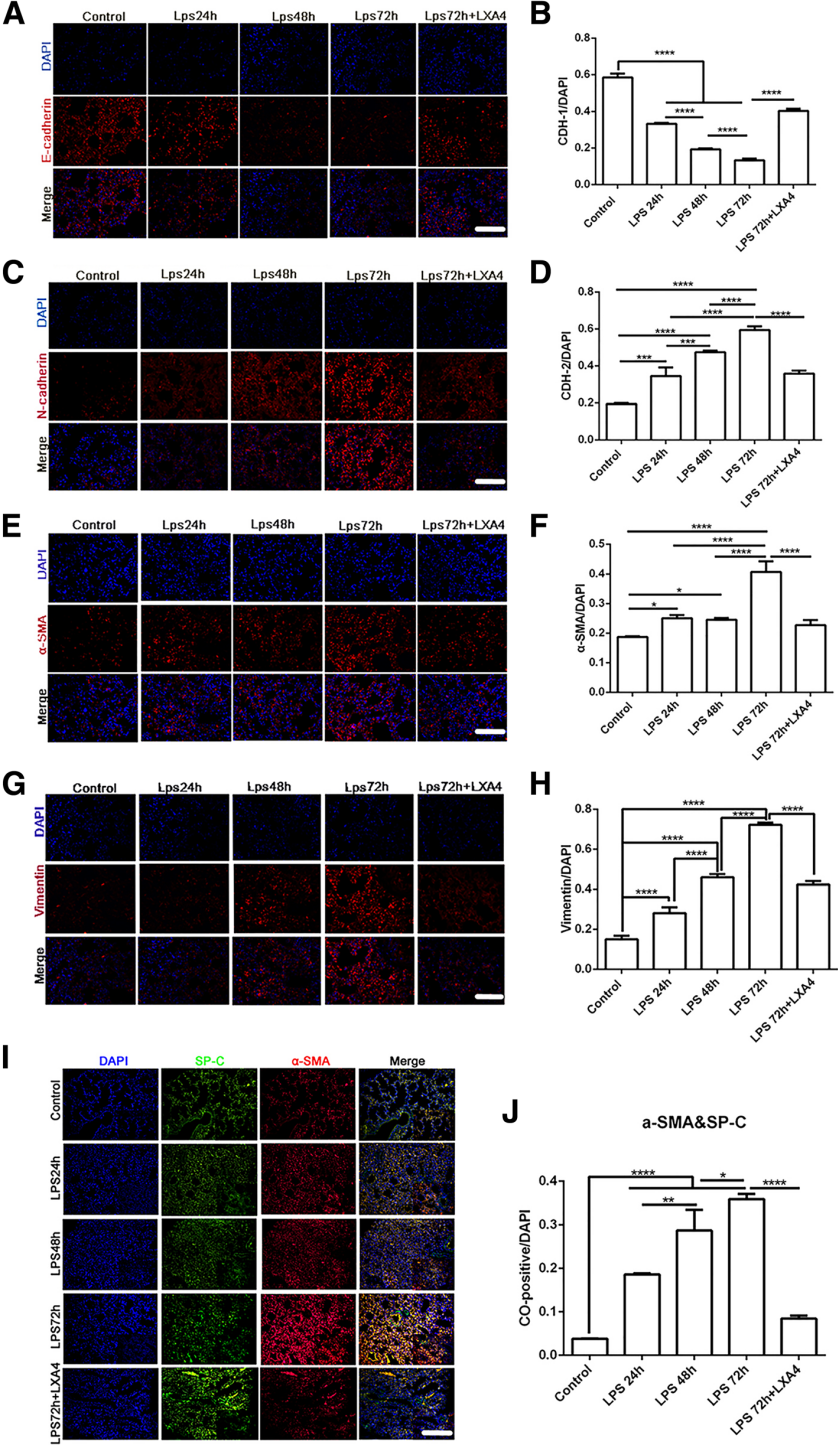
LXA4 inhibits the epithelial-mesenchymal transition (EMT) in LPS-induced lung injury. C57BL/6 J mice were intra-tracheal given N.S or LPS 10 mg/kg (for 24 h, 48 h or 72 h), with or without LXA4 1μg per mouse intraperitoneal injection. Immunofluorescence staining of lung specimens was shot by fluorescence microscope and calculated by positive goals compared to DAPI. **a**-**h**: Immunofluorescence staining of marker of EMT (× 400): E-cadherin(**a**-**b**), N-cadherin(**c**-**d**), α-SMA(**e**-**f**) and Vimentin(**g**-**h**). **i** and **j**: co-dying of SP-C and α-SMA. scar bar = 50 μm. All of the data was conducted in triplicate. Data were presented with means ± SD. n = 3. *P < 0.05, **P < 0.01, ***P < 0.001, ****P < 0.0001

To observe the process of EMT in the LPS-induced lung injury model, we performed immunofluorescence staining of the EMT markers including E-cadherin, α-SMA, N- cadherin and vimentin. We found that LPS reduced the expression of epithelial cell markers E-cadherin in a time dependent manner, while LXA4 promoted the expression of E-cadherin in the lung tissue (Fig. 4a, b). In contrast, LPS increased the expression of mesenchymal cell markers including N- cadherin, α-SMA and vimentin in a time dependent manner, but LXA4 down-regulated the expression of mesenchymal cell markers stimulated by LPS (Fig. 4c-h). To determine whether AT II cells undergo EMT process in LPS-induced lung injury, lung specimens SP-C (a type II cell marker) and α-SMA double staining immunofluorescence were observed. We found that SP-C/α-SMA double positive cells were increased after LPS treatment (Fig. 4i, j). However, treatment with LXA4 significantly decreased SP-C/α-SMA double positive cells in the intratracheal LPS murine model of ALI/ARDS (Fig. 4i, j).

### TGF-β_1_ induces EMT in HAT II cells

**Fig. 5:**
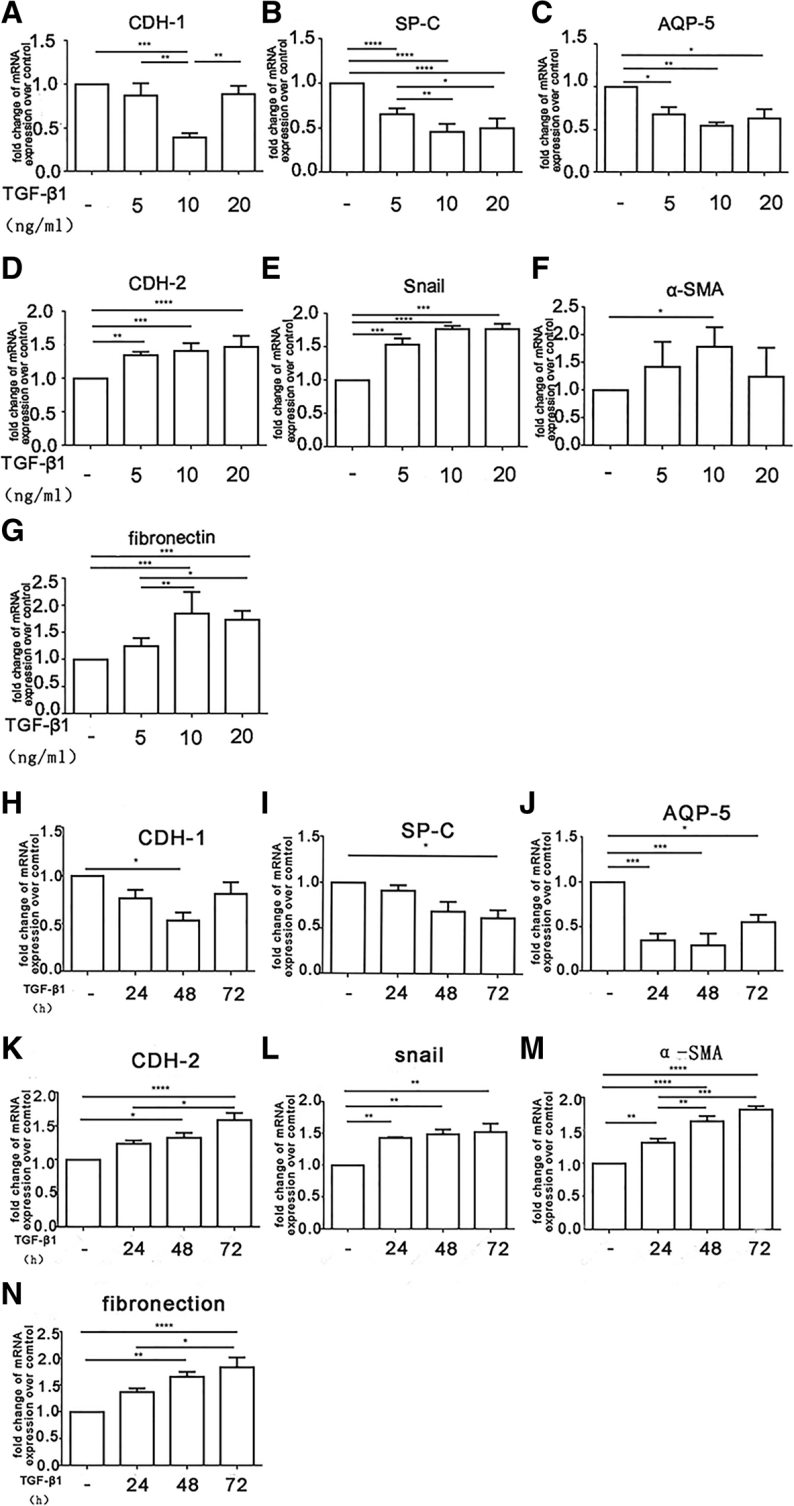
TGF-β_1_ induces EMT in primary human lung alveolar type II (HAT II) cells. HAT II cells were cultured as mentioned in the methods. **a**-**g**: HAT II cells were incubated with TGF-β_1_ (0 ng/ml, 5 ng/ml, 10 ng/ml, 20 ng/ml) for 48 h. **h**-**n**: HAT II cells were incubated with TGF-β_1_ 10 ng/ml for 0 h, 24 h, 48 h and 72 h. The expression of CDH-1, SP-C, AQP-5, CDH-2, snail, α-SMA and fibronectin were assessed by Real-time PCR. n = 4 for each culture condition, repeated using cells from 4 donors. Data were presented with means ± SEM, *P < 0.05, **P < 0.01, ***P < 0.001, ****P < 0.0001

To investigate if TGF-β_1_ could induce EMT in HAT II cells, HAT II cells were incubated with TGF-β_1_ (0 ng/ml, 5 ng/ml, 10 ng/ml, 20 ng/ml) for 48 h or with TGF-β_1_ 10 ng/ml for 0 h, 24 h, 48 h and 72 h. We found that the mRNA levels of epithelial markers, including CDH-1 (Fig. 5a), SP-C (Fig. 5b) and AQP-5 (Fig. 5c) in different TGF-β_1_ concentration groups, were all reduced by treatment with TGF-β_1_, and 10 ng/ml TGF-β_1_ group was lower than other groups. The mRNA levels of mesenchymal markers including CDH-2 (Fig. 5d), snail (Fig. 5e), α-SMA (Fig. 5f), and fibronectin (Fig. 5g), were promoted as the TGF-β_1_ concentration increased. There was no significant difference in the mRNA levels between treatment with 10 ng/ml and 20 ng/ml TGF-β_1_. After HAT II cells were treated with TGF-β_1_ 10 ng/ml for 0 h, 24 h, 48 h and 72 h, the mRNA expression of CDH-1 (Fig. 5h), SP-C (Fig. 5i) and AQP-5 (Fig. 5j) were inhibited by TGF-β_1_ at different time points, and the mRNA expression of CDH-1, AQP-5 at 48 h were lower than at other time points, while the mRNA expression of SP-C reached the lowest level at 72 h. However, the mRNA expression of mesenchymal markers, including CDH-2 (Fig. 5k), Snail (Fig. 5l), α-SMA (Fig. 5m), fibronectin (Fig. 5n) were higher at 72 h than other time points, but there was no significant difference in the mRNA levels between treatment with TGF-β_1_ for 48 h and 72 h. Based on these results, it is reasonable to establish the vitro EMT model with the concentration of TGF-β_1_ 10 ng/ml for 48 h.

### LXA4 inhibits the EMT induced by TGF-β_1_ in HAT II cells

**Fig. 6:**
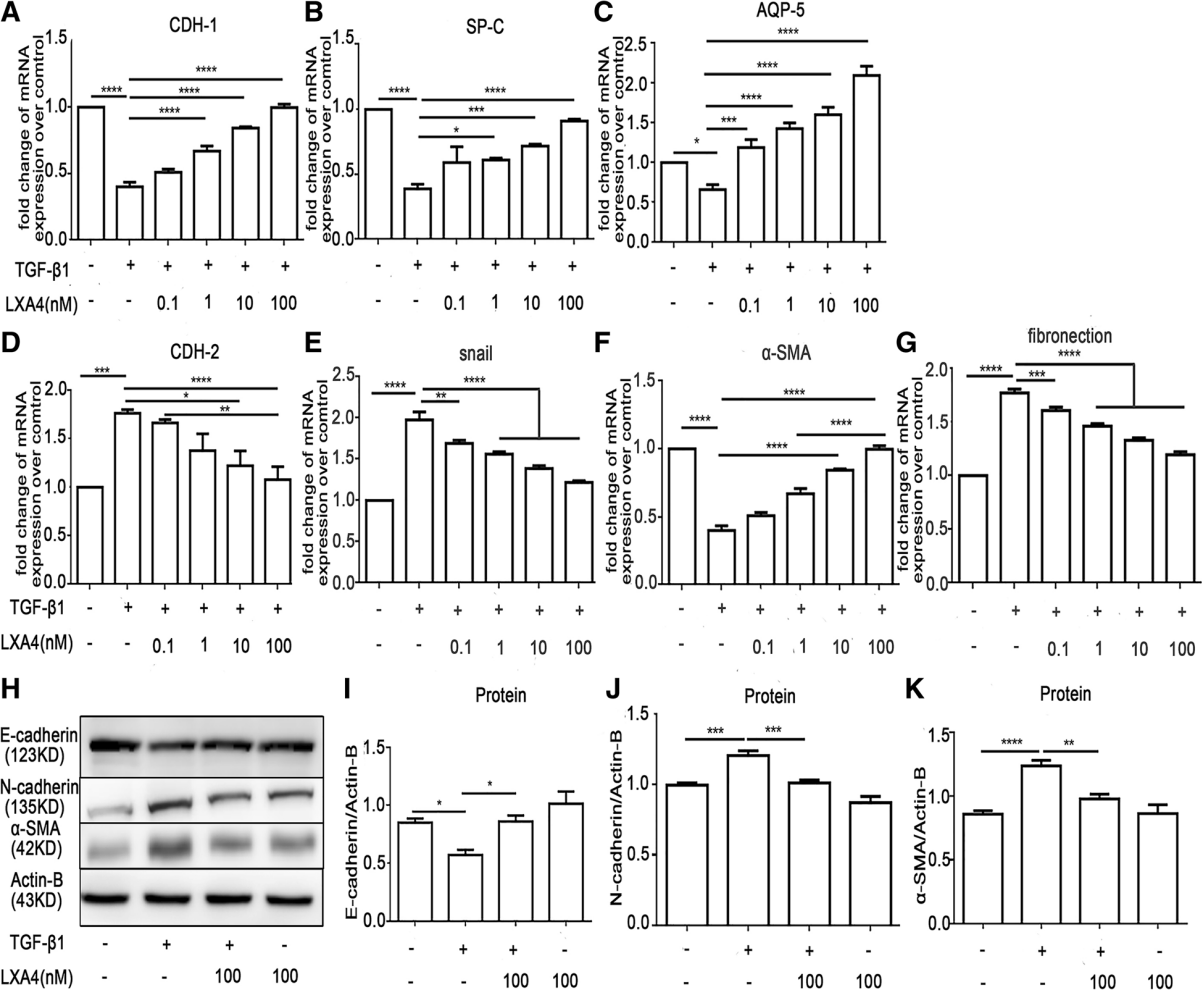
LXA4 inhibits TGF-β_1_ induced EMT in primary HAT II cells. HAT II cells were incubated with or without TGF-β_1_ 10 ng/ml for 48 h with or without LXA4 0.1 nM, 1 nM, 10 nM and 100 nM. **a**-**g**: the mRNA expression of CDH-1, SP-C, AQP-5, CDH-2, α-SMA and fibronectin. **h**-**k**: the protein expression level of E-cadherin, N-cadherin and α-SMA. n = 4 for each culture condition, repeated using cells from 4 donors. Data were presented with means ±SEM. *P < 0.05, **P < 0.01, ***P < 0.001, ****P < 0.0001

To investigate the effect of LXA4 on EMT induced by TGF-β_1_, Realtime-PCR and western blotting analyses were applied respectively. As shown in Fig. 6a-g, LXA4 promoted the mRNA expression of epithelial cell markers (CDH1, SP-C and AQP-5) in a does-dependent manner, while inhibiting the mRNA expression of mesenchymal cell markers, including CDH2, Snail, fibronectin and α-SMA in a does-dependent manner. Furthermore, the effects of LXA4 (100 nM) on the TGF-β_1_-induced CDH1 (E-cadherin), α-SMA, CDH2 (N-cadherin) protein expression of HAT II cells were confirmed by western blot (Fig. 6h-k).

### LXA4 inhibits TGF-β_1_-induced EMT in primary HAT II cells through activation of LXA4 receptor (ALX)

**Fig. 7:**
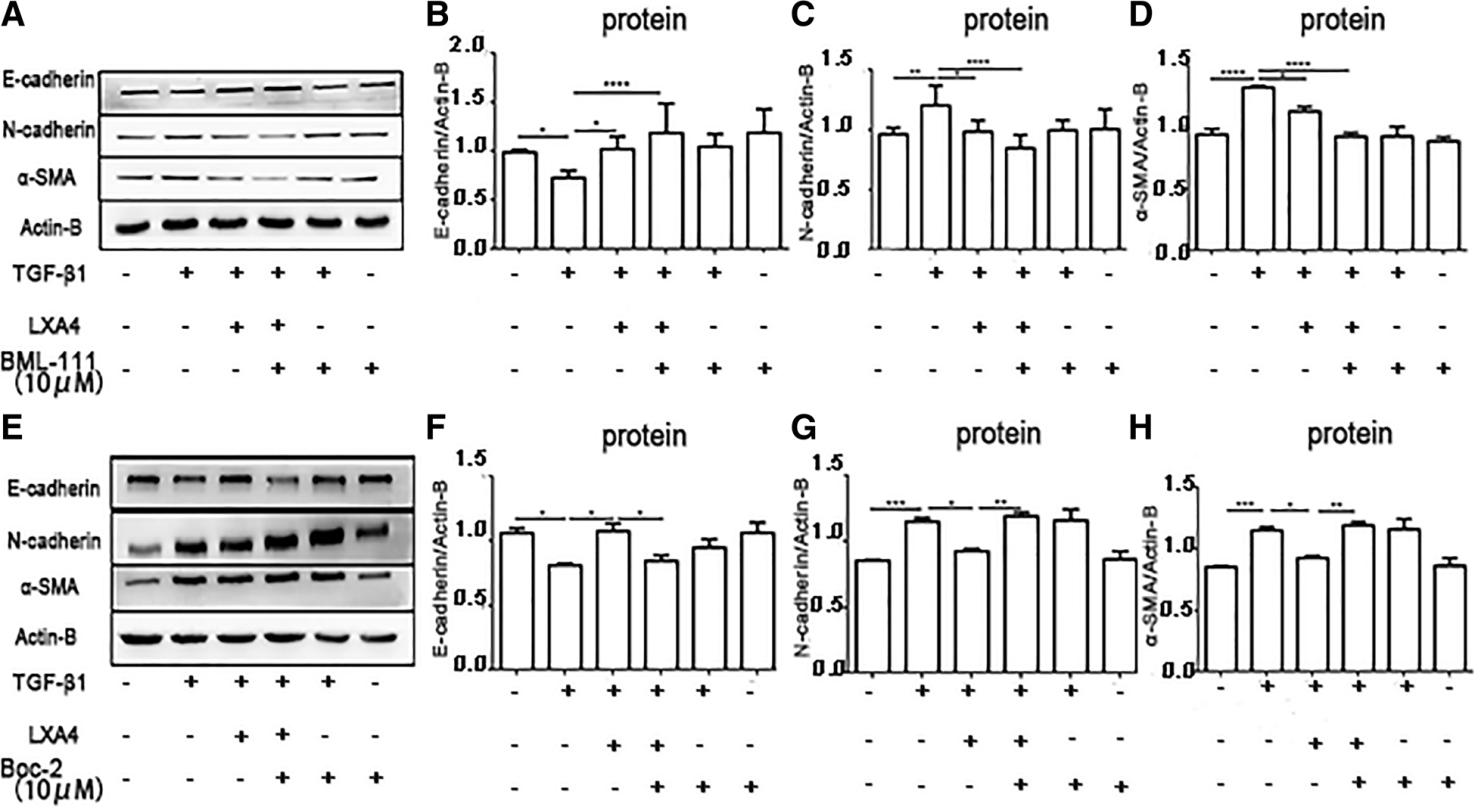
LXA4 inhibits TGF-β_1_-induced EMT in primary AT II cells through activation of LXA4 receptor (ALX). HAT II cells were pre-incubated with or without BOC-2 10μΜ or BML-111 10μΜ 30 min before TGF-β_1_ 10 ng/ml for 48 h with or without LXA4 100 nM. **a**-**d**: the effect of LXA4 on EMT was promoted by the pre-incubation of AT II cells with BML-111 (the LXA4 receptor agonist). **e**-**h**: the effect of LXA4 on EMT was abrogated by the pre-incubation of AT II cells with BOC-2(the LXA4 receptor antagonist). n = 4 for each culture condition, repeated using cells from 4 donors. Data were presented with means ±SEM. *P < 0.05, **P < 0.01, ***P < 0.001, ****P < 0.0001

To identify the involvement of ALX in LXA4 blockade of TGF-β_1_ induced EMT, HAT II cells were pre-treated with ALX ligands including BOC-2 10 μM (the LXA4 receptor antagonist) and BML-111 10 μM (the LXA4 receptor agonist) separately for 30 min. The effect of LXA4 on EMT was abrogated by the preincubation of HAT II cells with BOC-2 (Fig. 7a-d). While BML-111 promoted the effects of LXA4 on TGF-β_1_-induced EMT in HAT II cells (Fig. 7e-h). These results suggest that the effects of LXA4 on TGF-β_1_-induced EMT are mediated via activation of ALX.

### LXA4 reduces TGF-β_1_-induced EMT in primary HAT II cells partly through the SMAD and PI3K/AKT signaling pathway

**Fig. 8:**
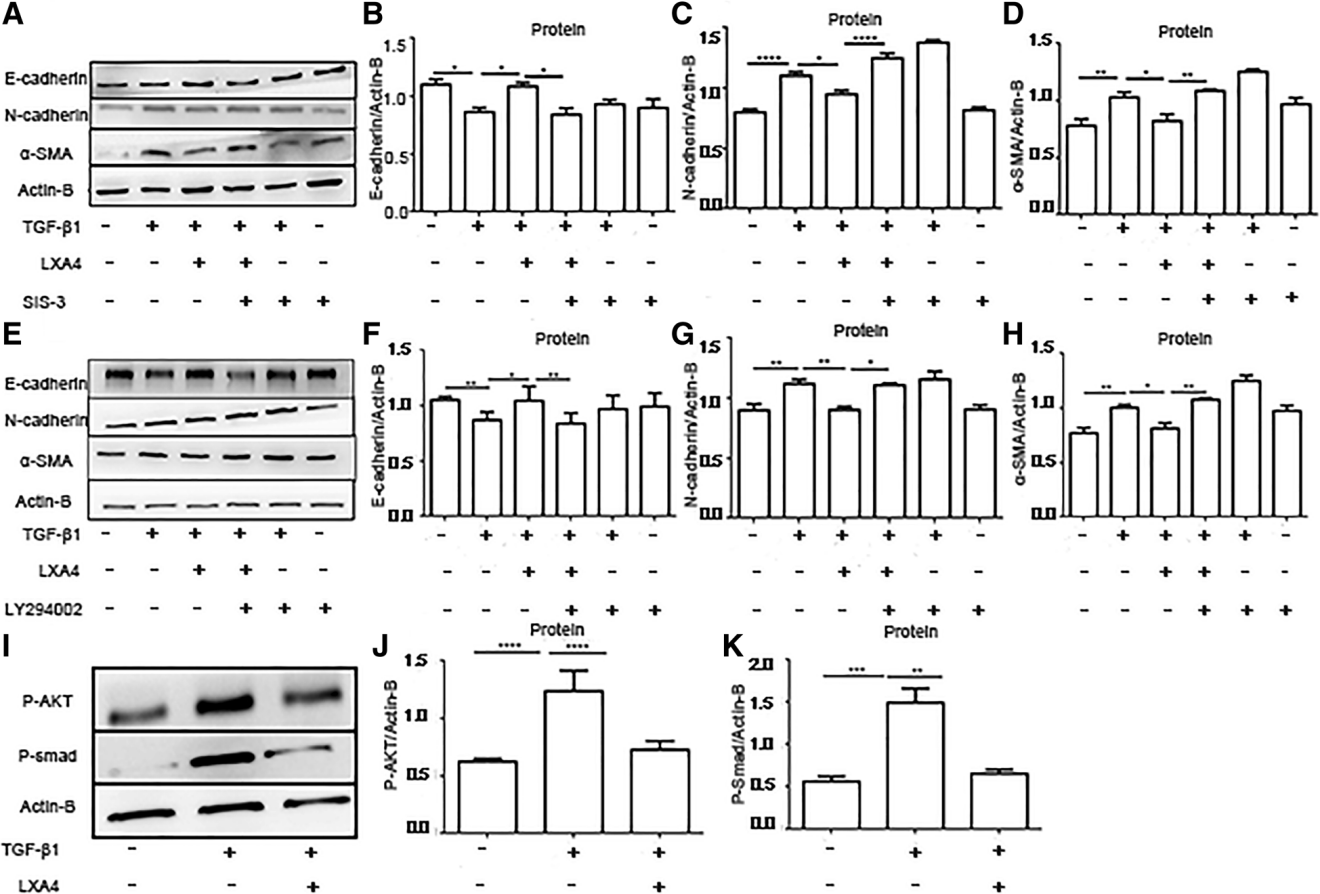
LXA4 reduces TGF-β_1_-induced EMT in HAT II cells partly through the SMAD and PI3K/AKT signaling pathways. HAT II cells were pre-incubated with 10 μM Sis3 (a specific Smad3 inhibitor), and 10 μM LY294002(PI3Kinhibitor) for 30 min prior to TGF-β_1_ 10 ng/ml for 48 h with or without LXA4 100 nM. **a**-**h**: Sis3 and LY294002 treatment abolished the inhibition of LXA4 on the EMT in AT II cells. **i**-**k**: LXA4 inhibited TGF-β_1_-induced the phosphorylation of AKT and Smad in primary AT II cells. n = 4 for each culture condition, repeated using cells from 4 donors. Data were presented with means ±SEM. *P < 0.05, **P < 0.01, ***P < 0.001, ****P < 0.0001

To confirm the involvement of the Smad2/3 and PI3K/Akt pathways in LXA4 blockade of TGF-β_1_-induced EMT in primary HAT II cells, HAT II cells were pre-treated with 10 μM Sis3 (a specific Smad3 inhibitor) and 10 μM LY294002 (PI3Kinhibitor) for 30 min prior to TGF-β_1_ and/or LXA4 administration. Sis3 and LY294002 treatment abolished the inhibitory effect of LXA4 on the EMT in HAT II cells (Fig. 8a-h). To further determine the activity of the PI3K/AKT and SMAD signaling pathways in primary HAT II cells stimulated by TGF-β_1_ after treatment with LXA4, the phosphorylation of AKT and Smad in HAT II cells were measured. The expressions of p-AKT and p-Smad were stimulated by TGF-β_1_ in primary HAT II cells and significantly downregulated by LXA4 (Fig. 8i-k). The agonist and antagonists had no effect on cell viability (Additional file 1: Figure S2).

## Discussion

Our study demonstrated that LXA4 alleviated lung injury via promoting type II alveolar lung epithelial cell proliferation, whilst inhibiting apoptosis and decreasing caspase-3 activation in an intratracheal LPS murine model of ALI/ARDS. In vitro, LXA4 reduced AT II cell apoptosis and promoted AT II cell proliferation induced by LPS. We also showed that LXA4 inhibited EMT in vivo and reduced TGF-β_1_ induced EMT in human primary type II alveolar epithelial cells. Furthermore, treatment with LXA4 receptor antagonist, Smad2/3 inhibitor and PI3K/AKT inhibitor abolished the inhibitory effect of LXA4 on the EMT in AT II cells, indicating that LXA4 can inhibit TGF-β_1_-induced EMT in primary AT II cells through the SMAD, PI3K/AKT signaling pathways and activation of LXA4 receptor (ALX).

The epithelial cell is a main target in the development of ALI/ARDS [21]. Injury of the alveolar epithelial cells (AT II cells) are acknowledged as critical hallmark of ARDS [22]. Timely repair of AT II cells is critical for restoration of lung function in ARDS. Inappropriate repair, such as EMT, can lead to disrupted barrier function and promote fibrogenesis [21]. Many studies reported that LXA4 exerts a protective effect on ALI in mice and on the airway epithelial cells [18, 23, 24]. Our previous study also showed that LXA4 alleviated inflammation and pulmonary permeability [18]. In order to investigate the potential mechanism of LXA4 in promoting resolution of ARDS, we previously demonstrated that LXA4 promoted lung epithelial repair and inhibited sFasL induced AT II cell apoptosis in vitro. In the present study we used an animal model of LPS-induced lung injury to confirm the previous results. We found that intratracheal instillation of LPS inhibited the proliferation of AT II cells and increased apoptosis of these cells. However, LXA4 restored the function of the epithelial barriers by reversing the inhibition of LPS on AT II cell proliferation and reducing apoptosis of AT II cells induced by LPS. In addition, LXA4 promoted primary AT II cell proliferation and reduced apoptosis induced by LPS [25, 26].

As a central role in the execution of the apoptotic program, caspase-3 is primarily responsible for the cleavage of poly (ADP-ribose) polymerase (PARP) during apoptosis [27, 28]. In our study, treatment with LPS in mice significantly increased TUNEL-positive AT II cells and cleaved caspase-3 expression in the lung tissue. However, LXA4 reduced LPS-stimulated cleaved caspase-3 expression and TUNEL-positive AT II cells at 24 h in lung tissue, indicating its anti-apoptotic effects in this murine model of lung injury.

Previous evidence in animal models of ARDS showed that pulmonary edema can happen only after the impairment of epithelium function [5, 29, 30]. Injury to the AT II cells activates apoptotic markers such as caspases-3, while some of the AT II cells undergo EMT which includes loss of their epithelial morphology as well as epithelial biomarkers and acquisition of a mesenchymal (fibroblast-like) cell phenotype [30–33]. Inflammation, which is one of the primary causes of ARDS, also results in EMT [33]. LPS was shown to induce EMT [32], while LXA4 could suppress EMT in proximal tubular epithelial cells, pancreatic cancer cells and hepatocarcinoma cells [34–36]. In our study, LPS induced EMT in a time dependent manner. We also demonstrated that LXA4 stimulated the expression of E-cadherin while inhibiting the expressions of mesenchymal cell markers including N-cadherin, vimentin and α-SMA in LPS induced lung injury. Furthermore, we also showed that AT II cells expressed more mesenchymal biomarkers (α-SMA), which was inhibited by treatment with LXA4 in the lung tissue. These data indicate that targeting the anti-EMT actions of LXA4 may be a therapeutic strategy for treating ARDS.

To confirm the result that LXA4 suppressed EMT in lung tissue, we investigated the effect of LXA4 on EMT in vitro. We showed that TGF-β_1_ induced EMT in primary human lung alveolar type II (HAT II) cells, while LXA4 inhibited TGF-β_1_ induced EMT in a concentration-dependent manner. In addition, LXA4 exerts its pro-resolving action through ALX (lipoxin receptor) [37]. In the present study, BOC-2 (ALX antagonist) reversed LXA4-suppressed EMT. Interestingly, BML-111(lipoxin receptor agonist), which was used in this study, promoted the effects of LXA4 on TGF-β_1_-induced EMT in primary human AT II cells. These data imply that LXA4 may act via activation of ALX.

Various studies have demonstrated underlying mechanisms involved in TGF-β_1_ induced EMT including the Smad signaling pathway and the PI3K/Akt signaling pathway [38, 39]. Our study suggests that inhibition of Smad3 and PI3K abolished the inhibitory effects of LXA4 on EMT in AT II cells, indicating that LXA4 inhibits EMT via the Smad and the PI3K/Akt signaling pathways. Indeed, in our study, LXA4 downregulated the phosphorylation of AKT and Smad induced by TGF-β_1_ in AT II cells.

## Conclusion

In conclusion, we have shown that LXA4 attenuates lung injury via stimulating epithelial cell proliferation, reducing epithelial cell apoptosis and inhibits EMT. In addition, LXA4 suppressed TGF-β_1_ induced EMT through the SMAD, PI3K/AKT signaling pathways and activation of LXA4 receptor (ALX). Our findings provide the evidence that targeting the pro-proliferaory, anti-apoptotic and anti-EMT actions of LXA4 may be a potential approach in developing an effective strategy for the treatment of ARDS. Further experiments are necessary to understand the basic mechanism underlying the anti-apoptotic effects of LXA4.

## Additional file

Additional file 1: **Figure S1**. LXA4 alleviates inflammation and pulmonary permeability in LPS-induced lung injury. A-E: HE staining of lung. F: injury score of lung. G: wet/dry ratio of lung. Data were presented with means ±SD. ***P<0.001, ****P<0.0001. n=3. **Figure S2**. The agonist and antagonists had no effect on cell vialibity. There is no significant of the agonist and antagonists on HATII cell vilibity. Data were presented with means ±SD. n=3. (DOCX 605 kb)

## Data Availability

The datasets used and/or analyzed during the current study are available from the corresponding author on reasonable request.

## Abbreviations

ALI: Acute lung injury
AQP-5: Aquaporin V
ARDS: Acute respiratory distress syndrome
AT II: Alveolar Type II
CDH-1: E-cadherin
CDH-2: N-cadherin
EMT: Epithelial-Mesenchymal Transition
HAT II: Human Alveolar Type II
LPS: Lipopolysaccharides
LXA4: Lipoxin A4
PCNA: Proliferating Cell Nuclear Antigen
SP-C: Surfactant protein C
TGF-β_1_: Transforming growth factor-β1
TUNEL: Terminal deoxynucleotidyl transferase-mediated dUTP-biotin nick End Labeling
α-SMA: α-Smooth muscle actin
