# Supplementary material for: Lipoxin A4 ameliorates lipopolysaccharide-induced lung injury through stimulating epithelial proliferation, reducing epithelial cell apoptosis and inhibits epithelial–mesenchymal transition
Source: Respir Res. 2019 Aug 22;20:192. doi: 10.1186/s12931-019-1158-z (PMC6704532; doi:10.1186/s12931-019-1158-z)
Supplement: Supplementary file 1 — Figure S1. LXA4 alleviates inflammation and pulmonary permeability in LPS-induced lung injury. A-E: HE staining of lung. F: injury score of lung. G: wet/dry ratio of lung. Data were presented with means ±SD. ***P<0.001, ****P<0.0001. n=3. Figure S2. The agonist and antagonists had no effect on cell vialibity. There is no significant of the agonist and antagonists on HATII cell vilibity. Data were presented with means ±SD. n=3. (DOCX 605 kb) [file 12931_2019_1158_MOESM1_ESM.docx]

**Material and methods:**

**Lung specimen**

HATII cells were isolated from lungs of grossly normal appearance after resection for lung tumor. All procedures in this study were carried out in accordance with approval from the local research ethics committees at the Second Affiliated Hospital of Wenzhou Medical University from May 2015 to December 2017. All patients gave written informed consent for the use of their tissue and clinical data for research purposes. Lung resection specimens alveolar type II cells were extracted from patients undergoing lung cancer resection. We used cells from 15 donors for ATII cell extraction who had normal lung function (7M:8F, mean age 60.5 years). Resected lung specimens were immediately examined in the operating theatre by a member of the surgical team. A portion of specimen without macroscopic pathology and not required for a diagnostic purpose was passed to the research team. This sample was immediately immersed in sterile 0.9% saline in a sealed container and transported on ice to the laboratory for processing. The sample was measured and superficially washed with 0.9% saline immediately on arrival at the laboratory.

**Primary human alveolar type II cells isolation and culture.**

Primary human alveolar type II (HATII) cells were extracted according to methods described previously^1^. Briefly, Wash the tissue (lung section) with sterile saline to remove blood and debris from the surface. Place the tissue into a fresh Petri dish ready for trypsinisation. Instill the trypsin solution (10-15 ml/5cm3 piece, Gibco 25300) into the lung tissue in exactly the same way as the saline lavage. Place the covered Petri dish into a 37 °C incubator for 15 min. Repeat the procedure twice more to give a total trypsinisation period for 45 min. Chop the tissue finely into 1- 2 mm3 in the presence of FCS (10ml/5cm3 piece) and DNase I (250 µg/ml HBSS; Sigma DN25, HB9394). Shake the minced tissue suspension vigorously by hand for 5 min to enhance type II cell recovery. Filter the tissue suspension through a large gauge mesh (400-500 µm) and then a 40 µm cell filter (BD Biosciences) to remove undigested tissues and debris from the enzymatically-released epithelial cells, which pass through the filter. Centrifuge the filtrate, containing mostly single cells, at 300g at 12°C for 7 min. Suspend the cell pellets in 50-100 ml 50% DCCM-1 and 50% HBSS containing 100 µg/ml DNase I. Plate the resuspended cell suspension into either T-75 or T-175 culture flasks and incubate at 37°C for 1.30 h to enable any contaminating macrophages to adhere. Remove the media containing the nonadherent type II cell-enriched cell population and centrifuge at 300g at 12°C for 7 min. Resuspend the cell pellet in 3 ml of red cell lysis buffer and incubate for 3 min. Add enough HBSS to make up the volume and centrifuge the filtrate at 300g at 12°C for 7 min. Resuspend the cell pellet in a known small volume of (5 ml) 10% DCCM1(Biological Industries Ltd. Kibbutz Beit-Haemek, Israel) and make up the volume 10 ml. Count the epithelial cells using as haemocytometer by phase contrast microscopy. Filter again if clumps were found. Prepare a cytospin for alkaline phospatase staining if required. Add 10% DCCM-1 so that the cells are 1 x 106 epithelial cells/ml and plate onto collagen-coated plates; 1 x 106 /well of a 6 well plate (for western blot studies), 0.5 ×10^6^ /well of a 24 well plate(for PCR and Flow Cytometry Analysis) and 0.4 x 105 / well of a 96-well plate (make media up to 1ml/well, 500µl/well and 200 µl/well, respectively for cell proliferation assay). After 24 h remove the media and nonadherent cells. Do not wash, leave remaining loosely attached cells and apply fresh 10% DCCM-1 medium. After another 16-24 h, remove medium and wash off remaining loose cells with HBSS. Apply fresh complete medium. The cells from a confluent monolayer within 3 days of plating. Average yields of primary human alveolar type II cells were 30.2 million cells per resection with an average purity of 92%. Cells were tested for primary human alveolar type II (HAT II) cell phenotype by alkaline phosphatase staining, lysotracker lamellar body staining and by PCR expression of surfactant protein C—a type II cell marker with negative expression of aquaporin V (a type I cell marker) (data not shown). 0.5 Million cells were seeded onto 24 well plates and grown for 3 days in DCCM-1 (Biological Industries Ltd. Kibbutz Beit-Haemek, Israel) media supplemented with 10% fetal calf serum (FCS). Before stimulation cells were serum starved overnight (0.1% FCS) and stimulated in medium containing 0.1% FCS for 24 hours.

Measurement of Lung Water Content—Wet/Dry Weight Ratio

To quantify the magnitude of pulmonary edema, we evaluated the wet weight to dry weight (wet/dry) ratio of the lung. Portions of the harvested wet lower lobes of the right lung were weighed, then placed in an oven for 48h at 60°C until weight was no longer changed. The dry lungs were weighted and the wet-to-dry (W/D) ratio was calculated.

**Histological analysis of lung tissues**

For histological examinations, the middle lobe of the right lung of each animal was fixed with 10% paraformaldehyde for 24 h, embedded in paraffin wax, sectioned, and stained with haematoxylin for 5min, incubated in Scott’s tap water (tap water with a few drops of 1 M sodium hydroxide) for 5 min and stained with alcoholic eosin solution for 1min. They were washed by tap water for 2 min after every step. Then tissues were differentiated by immersion in 1% hydrochloric acid-ethanol each for 20s. Tissues were then mounted by inversion onto glass slides dotted with Gel/Mount for conventional morphological evaluation under light microscope (Nikon eclipse 90i, Tokyo, Japan).. Lung injury scores were quantified by an investigator who was blinded to the treatment groups using an established histopathological scoring system.

**BRUD U cell proliferation assay**

4×10^5^ cells/ml (HATII cells) were seeded into a 96 well culture dish. Brud U Label was added and cells were incubated with boc-2 10μM，BML-111 10μM, LY294002 10μM and SIS-3 10μM. After 24 hours culture, Brud U incorporation was assessed according to manufacturers’ instructions (BRUD U Cell Proliferation Assay, Promega, UK).

**Immunofluorescence**

The right lung of each mouse was collected and cut into equal slices; they were fixed in 10% paraformaldehyde for 24 h. Then, they were embedded in paraffin. Specimens were cut in 5 μm serial sections and deparaffinized with xylene and rehydrated with ethanol and water, then were placed in a pressure cooker with citrate buﬀer, pH 6.0, for 1 min after reaching boiling temperature to retrieve antigenic sites masked by formalin fixation. Sections were incubated with antibody to SP-C, α-SMA, E-cadherin, N-cadherin and vimentin all in 1:100 dilution or with the pre-immune serum as a negative control stain overnight at 4 °C, and subsequently were incubated for 1 h at room temperature with the goat anti-secondary antibodies: Alexa Fluor 488 or Alexa Fluor 594 (ab150077, ab150120,ab150080) and DNA with 40,6 diamidino-2-phenylindole (DAPI; included in fluoroshield mounting medium, Sigma-Aldrich, F6057). Images were obtained using a 5000B Leica microscope equipped with a charge-coupled device camera (Retiga 200R) interfaced with Q-Capture Pro software (Q Imaging). Images were globally adjusted to optimize contrast and brightness, and composed using Image J. The quantitation of them were calculated by positive/DAPI.

**Results**

**Figure.S1 LXA4 alleviates inflammation and pulmonary permeability in LPS-induced lung injury. A-E: HE staining of lung. F: injury score of lung. G: wet/dry ratio of lung. Data were presented with means ±SD. ***P<0.001, ****P<0.0001. n=3.**


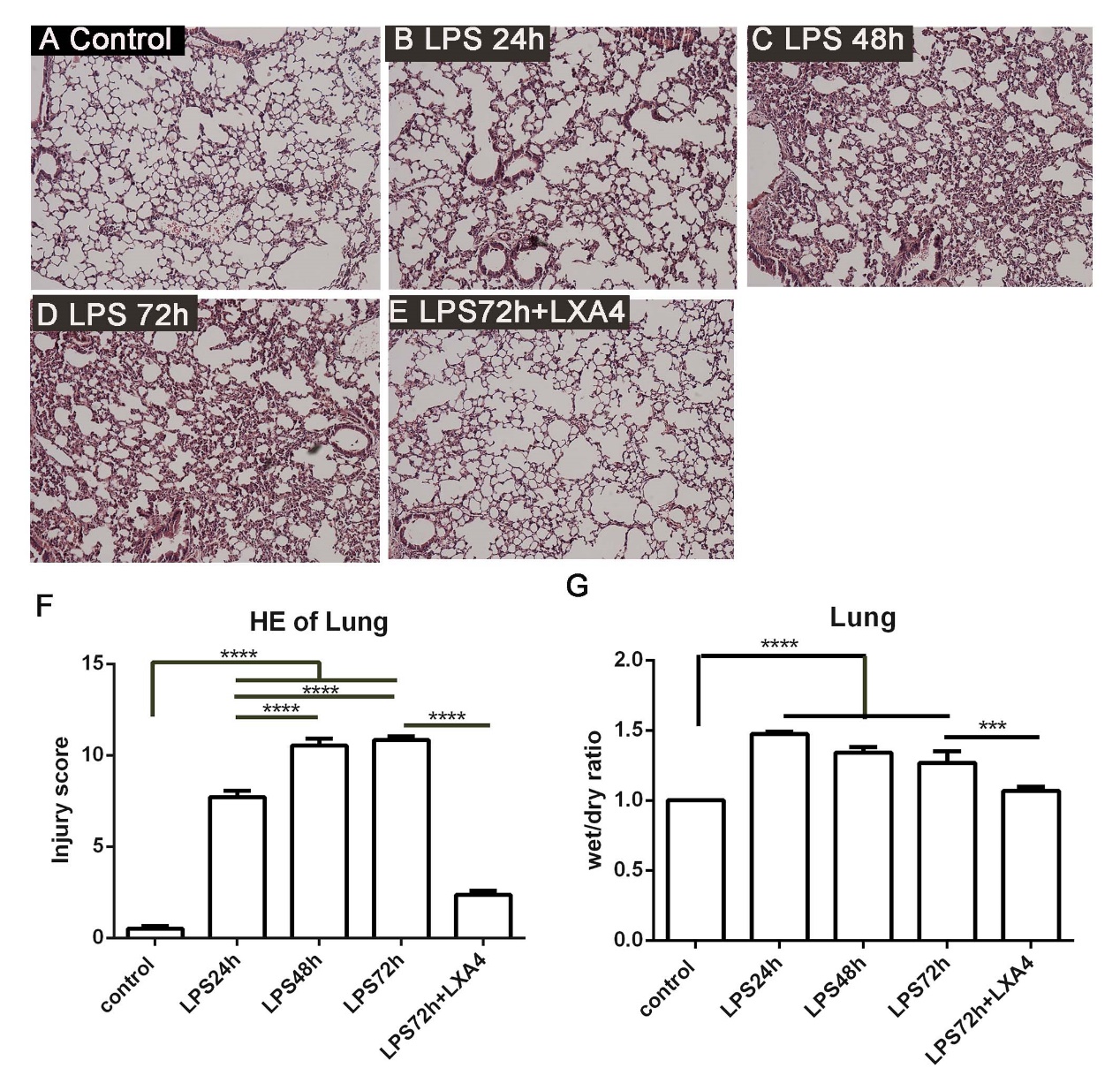


**Figure.S2 The agonist and antagonists had no effect on cell vialibity. There is no significant of the agonist and antagonists on HATII cell vilibity. Data were presented with means ±SD. n=3.**





**Reference**

1 Zheng, S. *et al.* Lipoxin A4 promotes lung epithelial repair whilst inhibiting fibroblast proliferation. *ERJ Open Res* **2**, doi:10.1183/23120541.00079-2015 (2016).
